# A Multidisciplinary Approach to Tracheoesophageal Fistula Repair in a Newborn: A Case Report

**DOI:** 10.7759/cureus.51359

**Published:** 2023-12-30

**Authors:** Varun N Thawkar, Karuna Taksande

**Affiliations:** 1 Anaesthesiology, Jawaharlal Nehru Medical College, Datta Meghe Institute of Higher Education & Research, Wardha, IND

**Keywords:** surgical repair, 2d echo, anesthetic management, lower segment cesarean section, trachea-oesophagal fistula, neonatal emergency

## Abstract

This case report details the emergency management and successful surgical repair of a tracheoesophageal fistula (TEF) in a newborn delivered by lower segment cesarean section. Despite immediate crying after birth, the neonate's distress was evident, with an Apgar score of 4, prompting an urgent referral to the Neonatal ICU (NICU). Diagnostic investigations, including ultrasonography and two-dimensional echocardiography (2D Echo), revealed associated anomalies, such as a patent ductus arteriosus, arterial septal defect, and a TEF. An anaesthetist was urgently involved due to postnatal desaturation, leading to challenging intubation and surgical repair performed under general anaesthesia, which involved separating the trachea from the oesophagus. Postoperative imaging confirmed the successful closure of the fistulous connection. This case highlights the significance of prompt diagnosis, collaborative management, and surgical intervention in optimising outcomes for neonates with complex congenital anomalies like TEF.

## Introduction

Congenital anomalies, particularly tracheoesophageal fistula (TEF), pose significant challenges in the neonatal period, demanding swift diagnosis and intervention to optimise outcomes [1]. A TEF represents a rare malformation characterised by an abnormal connection between the trachea and oesophagus, often associated with other congenital anomalies involving the cardiovascular and respiratory systems [2]. The incidence of TEF is estimated to be one in 2,500 live births, making it a relatively uncommon but clinically significant condition [3]. A TEF can manifest as part of the VACTERL association, a cluster of congenital anomalies involving vertebral, anal, cardiac, tracheal, oesophagal, renal, and limb abnormalities [4]. While the aetiology of TEF remains multifactorial, genetic factors and disruptions in fetal development contribute to its pathogenesis [5].

Early diagnosis of a TEF is crucial for initiating prompt and appropriate management. Clinical presentations may vary, with neonates often demonstrating signs of respiratory distress, cyanosis, and feeding difficulties shortly after birth. Diagnostic tools, such as ultrasonography (USG) and echocardiography (Echo), aid in identifying associated anomalies and guiding therapeutic decisions [6]. Surgical repair remains the mainstay of the treatment for a TEF, necessitating collaboration between neonatologists, pediatric surgeons, and anaesthetists. The successful separation of the trachea and oesophagus is paramount to ensuring proper respiratory and alimentary function. Anaesthetic management in neonates with a TEF requires careful consideration of the associated cardiovascular anomalies and potential difficulties in securing a patent airway [7]. This case report illustrates the complexity of managing a neonate with a TEF, emphasising the need for a comprehensive approach that integrates diagnostic precision, anaesthetic expertise, and surgical intervention.

## Case presentation

A newborn delivered through a lower segment cesarean section initially cried but garnered concern when assigned an Apgar score of 4. This distress prompted an emergency referral to the Neonatal ICU (NICU) for further evaluation. Despite a lack of obvious historical data, the physical examination revealed a hypoxic and cyanosed infant, prompting urgent diagnostic measures.

**Figure 1:**
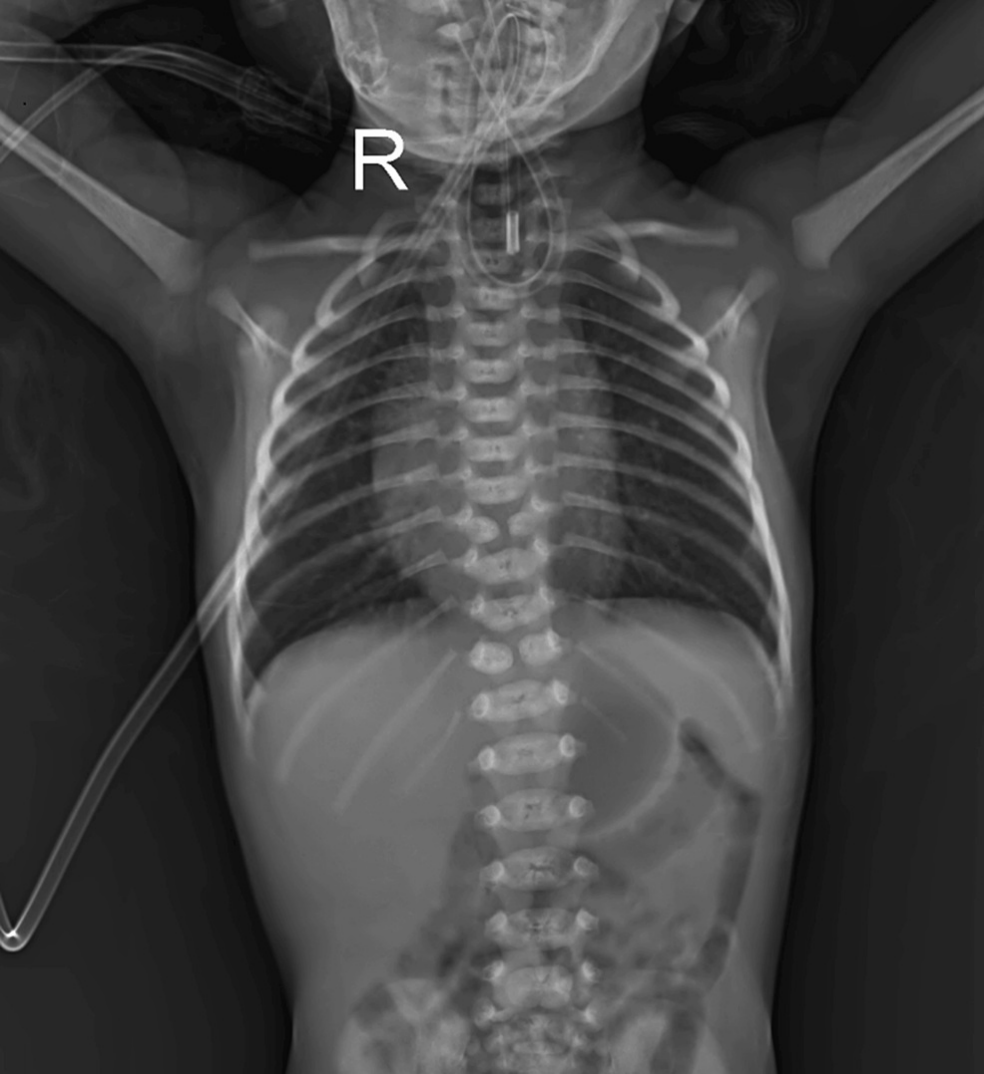
Preoperative X-ray imaging confirmed the diagnosis of a tracheoesophageal fistula

Upon admission to the NICU, diagnostic investigations were initiated to uncover the underlying cause of the distress. USG displayed a slightly raised echotexture of the bladder wall. Further, a two-dimensional (2D) Echo revealed the presence of a tiny patent ductus arteriosus with a left-to-right shunt, an arterial septal defect, and evidence of a TEF. This complex cardiac and respiratory anomaly necessitated immediate intervention. Preoperative X-ray imaging confirmed the diagnosis of a TEF (Figure 1).

**Figure 2:**
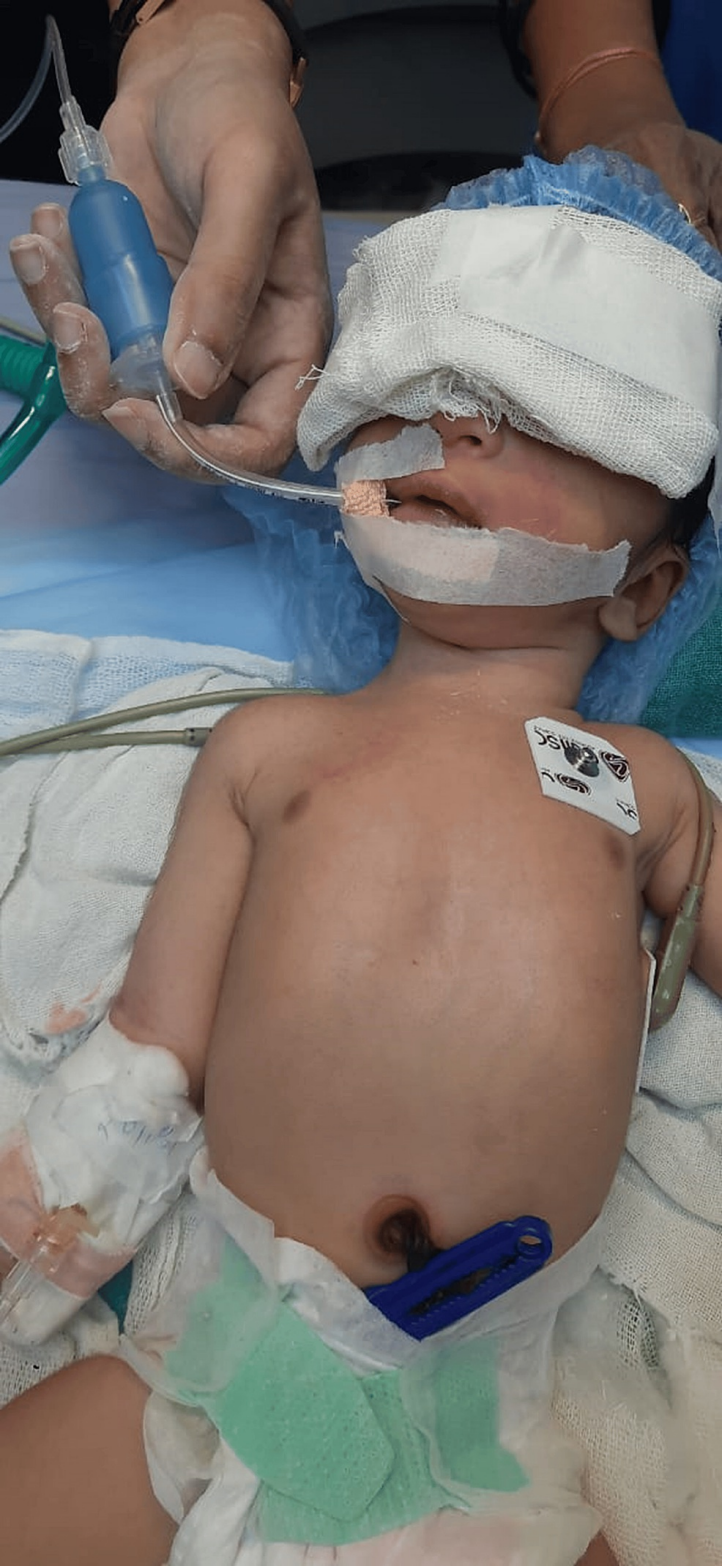
Successful intubation

Recognising the situation's urgency, an anaesthetist was urgently called to assess and manage the desaturation observed in the newborn. The initial attempts at intubation proved challenging, requiring a switch to a straight miller blade for a successful third attempt at securing a patent airway using an endotracheal tube Figure 2. All procedures were conducted with strict adherence to aseptic precautions to mitigate the risk of infection.

**Figure 3:**
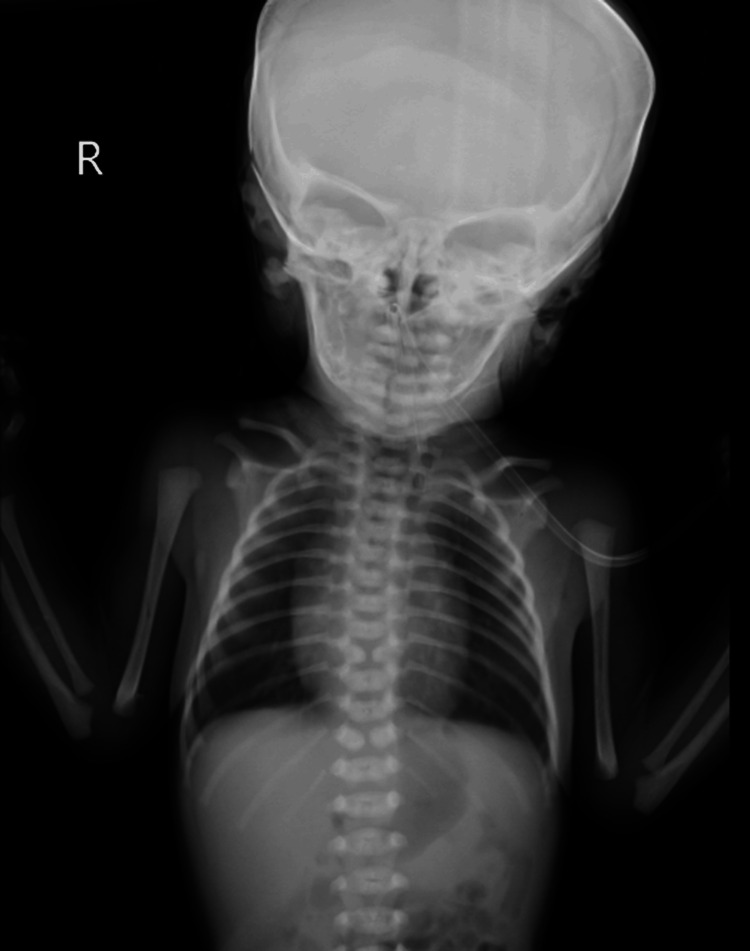
Postoperatively, X-ray imaging confirmed the successful separation of the trachea from the oesophagus

The diagnosis of a TEF prompted a decision for surgical repair under general anaesthesia. A low collar incision was meticulously made for access, and the trachea was carefully separated from the oesophagus. Esophagectomy was performed to address the anatomical defect. Postoperatively, X-ray imaging confirmed the successful separation of the trachea from the oesophagus, with no evidence of bleeding (Figure 3).

Following the procedure, the infant was kept nil per oral for four hours to facilitate recovery. Continuous monitoring in the NICU ensured the stabilisation of vital signs. The successful collaboration between neonatologists, anaesthetists, and surgeons facilitated a comprehensive approach to managing a neonate's complex presentation with a TEF.

## Discussion

The presented case underscores the intricate challenges of managing a TEF in the neonatal period. A TEF, often part of the broader spectrum of congenital anomalies, demands a multidisciplinary approach for accurate diagnosis, timely intervention, and comprehensive postoperative care. The incidence of TEF, estimated at one in 2,500 live births, highlights its rarity [8]. While the exact aetiology remains elusive, the condition is often associated with genetic factors and disruptions in fetal development [9]. The neonate, in this case, exhibited distress shortly after birth, emphasising the importance of recognising clinical cues indicative of a TEF, such as respiratory distress and feeding difficulties.

Diagnostic modalities, including USG and Echo, played a pivotal role in characterising the extent of the anomalies associated with the TEF [7]. Identifying a patent ductus arteriosus, arterial septal defect, and the fistulous connection between the trachea and oesophagus guided the subsequent management strategy. This highlights the significance of advanced imaging techniques in delineating the anatomical complexities often associated with a TEF [10]. The collaboration between neonatologists and anaesthetists was crucial in managing the immediate postnatal desaturation observed in the newborn. The challenges encountered during intubation, necessitating a change in blade type, underscore the need for adaptability in anaesthetic approaches when dealing with complex neonatal cases [11]. The successful intubation and subsequent surgical repair under general anaesthesia indicate the meticulous planning and execution essential in such scenarios.

Surgical intervention, the cornerstone of TEF management, involved a low-collar incision, separation of the trachea from the oesophagus, and esophagectomy. Postoperatively, contrast imaging confirmed the successful closure of the fistulous connection without evidence of bleeding, validating the efficacy of the surgical approach [12]. The comprehensive care provided to the neonate postoperatively, including nil per oral status and close monitoring, contributed to stabilising vital signs. This emphasises the significance of postoperative care in preventing complications and ensuring a smooth recovery.

## Conclusions

In conclusion, the presented case exemplifies the successful management of a neonate with a TEF, a complex congenital anomaly with potential life-threatening implications. The collaborative efforts of neonatologists, anaesthetists, and surgeons were pivotal in swiftly diagnosing the condition, addressing immediate postnatal distress, and executing a meticulous surgical repair. Integrating advanced diagnostic tools, such as USG and Echo, played a crucial role in characterising associated anomalies. Challenges faced during anaesthetic intervention underscore the need for adaptability and expertise in neonatal care. The surgical repair, involving the separation of the trachea and oesophagus, was successful, as confirmed by postoperative imaging. Close postoperative monitoring and adherence to a structured care plan facilitated the stabilisation of vital signs. This case underscores the importance of a comprehensive, multidisciplinary approach in addressing the complexities of TEF, emphasising the need for continued research to refine diagnostic and therapeutic strategies for improved outcomes in neonates with congenital anomalies.
